# Medical students’ perceptions of gamified design elements in a self-directed radiology department visit

**DOI:** 10.1186/s12909-026-10460-y

**Published:** 2026-09-24

**Authors:** Sultan R. Alharbi

**Affiliations:** Department of Radiology and Medical Imaging College of Medicine, King Saud University, Riyadh, Saudi Arabia

**Keywords:** Gamification, Medical education, Radiology education, Program evaluation, Self-directed learning, Undergraduate education, Student perceptions

## Abstract

**Background:**

Gamification in medical education has typically meant adding badges, points, or leaderboards to existing content. This paper reports medical students’ perceptions of five items evaluating a self-directed radiology department visit built around a themed storybook, a sticker system, a limited-access ID/passport, and a narrative “Kingdom” theme, alongside an overall rating of gamified understanding.

**Methods:**

In this single-centre, cross-sectional observational survey, third-year medical students enrolled in RAD366 at King Saud University who had completed the Radiology Kingdom activity during the 2025–2026 academic year were invited to anonymously report their perceptions of its gamified design elements. Of approximately 270 eligible students, 112 submissions were received (submission rate approximately 41.5%); after exclusion of one probable duplicate, 111 responses were analysed (analytic response rate approximately 41.1%). Four items assessed specific design features and one assessed a global outcome (students’ perceived understanding); differences across items were tested using a Friedman test with Bonferroni-corrected post-hoc comparisons, and between-group comparisons used Mann-Whitney U and Kruskal-Wallis tests. Internal consistency was assessed using Cronbach’s alpha. The questionnaire was administered under Institutional Review Board approval (No. R26-IRB-510) and included an explicit consent statement.

**Results:**

Cronbach’s α across the five items was 0.841, indicating substantial inter-item correlation; because the items assess conceptually distinct constructs, this coefficient was not interpreted as evidence of unidimensionality or instrument validity. A Friedman test identified a significant difference across items (χ²(4) = 32.353, *p* < 0.001): the ID/passport received significantly lower ratings than the other four items (all *p* ≤ 0.001, Bonferroni corrected), while no significant pairwise differences were detected among the Kingdom theme (4.63), gamified understanding (4.60), storybook (4.58), and sticker system (4.52) ratings. Female students gave significantly higher ratings than male students on the composite score (4.64 vs. 4.33; *p* = 0.023) and on three of five items, an exploratory finding. No significant difference in ratings was detected across visit months or prior radiology exposure groups.

**Conclusions:**

In this single-centre, observational survey, students reported highly positive perceptions of the storybook, Kingdom theme, sticker system, and gamified elements’ perceived contribution to their understanding, whereas the ID/passport item received significantly lower ratings; because the five items assessed different constructs, this difference reflects differences in student ratings rather than evidence of the comparative educational effectiveness of individual intervention components. These findings demonstrate the feasibility of implementing a resource-modest, narrative-based, self-directed clinical immersion activity within a busy radiology department, rather than evidence that gamification itself improved engagement, understanding, or learning, since the uncontrolled design does not support causal or comparative-effectiveness conclusions. The intervention offers a practical, replicable model for similar self-directed department visits that warrants evaluation in controlled, multi-institutional studies incorporating objective measures of learning and retention.

**Supplementary Information:**

The online version contains supplementary material available at https://doi.org/10.1186/s12909-026-10460-y.

## Introduction

During informal faculty discussions about the Radiology Kingdom materials, some colleagues described the visual style of the storybook—with its cartoon knights, superhero technologists, character stickers, and passport styled as an official travel document—as “childish.” Such reactions are not unusual in medical education, where a serious aesthetic is often assumed to be a prerequisite for rigorous learning. However, aesthetic impressions alone cannot determine whether an educational intervention achieves its intended educational objectives; that question requires empirical evidence rather than subjective aesthetic judgement. This study therefore reports how 111 medical students who completed the Radiology Kingdom activity evaluated its gamified design elements using a structured questionnaire.

Gamification in education has been defined as applying game-design elements in non-game contexts to enhance motivation and engagement [1]. A meta-analysis of 26 studies found significant positive effects on learning performance and motivation when gamification elements aligned with learning objectives rather than superficial decoration [2], and serious games in medical education have been associated with improved knowledge acquisition, engagement, and satisfaction [3]. A systematic review of health professions education reached a similar conclusion but noted that few studies explain the mechanisms underlying specific game attributes, calling for research on which individual elements drive which outcomes [4]. Most published studies evaluate an activity as a whole; this study reports on five items measured simultaneously in the same students, treated as distinct measures rather than assumed to form one construct.

The Radiology Kingdom was designed by the author, prior to any formal gamification taxonomy, to address three practical problems in delivering a self-directed radiology department visit to students who had not previously visited the department: an exposure gap widely noted in undergraduate radiology curricula, where radiology remains underrepresented despite its central role in modern healthcare [5]; how to manage large student numbers in a functioning clinical environment without disrupting patient care; and how to make the diverse professionals working within radiology — technologists, nurses, PACS staff, radiologists — visible and memorable to students. Three design responses followed; whether they achieved their intended functions, individually, is the empirical question this paper addresses.

The first response was narrative. Each imaging modality was assigned a named hero character whose name encodes a defining feature of that modality — for example, Lady Magnetica, representing MRI, whose name is intended to cue the absence of ionising radiation. This design was intended to draw on elaborative encoding, the principle that associating new information with meaningful, distinctive cues deepens memory traces [6]. Whether it does so in practice is a testable hypothesis that this study’s design does not directly address, and is returned to in the Discussion. A full description of the character set and storybook narrative is available from the author on request. The kingdom setting itself was chosen to reflect radiology’s layered historical development — new modalities added across decades (X-ray in 1895, ultrasound from the 1950s, CT in 1972, MRI in the 1980s) rather than arriving all at once.

The second response addressed crowd management: only 15 physical passports were available at a time, collected personally from the radiology department secretary, which limited the number of students in the department at any moment without imposing a fixed schedule. This scarcity was intended to function, in gamification terms, as a limited-access mechanic [7]. Whether students actually experienced the passport this way — as opposed to as an administrative requirement — was not directly measured in this study and is considered as one of several possible explanations in the Discussion.

The third response addressed navigation in a large and unfamiliar clinical environment by removing the guide entirely. Signs, coloured floor trails, and a map in the storybook guided students independently through the department, with no tutor leading the group. This design draws on self-directed learning theory, in which learners take primary responsibility for planning and carrying out their own learning experience [8], and on experiential learning theory, which holds that direct, concrete experience is the basis for durable knowledge construction [9]. It may also encourage additional student-initiated interactions with staff: students who cannot find a unit must ask for directions.

This paper reports students’ ratings of five items in the gamification and instructional-design domain of a post-visit questionnaire: four items assessing specific design features (storybook, sticker system, ID/passport, Kingdom theme) and one global outcome item (students’ perceived understanding), following the first full year of the activity’s implementation. The aim is descriptive: to report how these ratings compared with one another, whether they varied by participant characteristics, and whether they varied by time of year — without assuming in advance that the five items measure a single construct, or that any observed pattern demonstrates a validated design taxonomy.

## Methods

### Study design and setting

A cross-sectional questionnaire-based study was conducted at the Department of Radiology and Medical Imaging, King Saud University Medical City, Riyadh, Saudi Arabia — a tertiary referral centre performing over 1,000 radiology examinations daily — during the 2025–2026 academic year.

### The radiology kingdom activity

The Radiology Kingdom was designed and implemented by the author as a new component of RAD366, the third-year radiology module at King Saud University. Prior to this activity, the module included no scheduled visit to the radiology department. The activity was designed to give students structured, task-based exposure to the full professional team within the department while managing the operational constraints of a busy clinical environment.

Crowd management was addressed through a passport system: only 15 physical passports — styled as official travel documents to the Radiology Kingdom — were available at any time, collected personally from the radiology department secretary. Students kept their passport for their assigned visit week and returned it upon completion. This limited simultaneous student numbers without imposing a fixed timetable, preserving departmental workflow while creating a limited-access mechanic: only 15 students could be in the Kingdom at once.

**Fig. 1:**
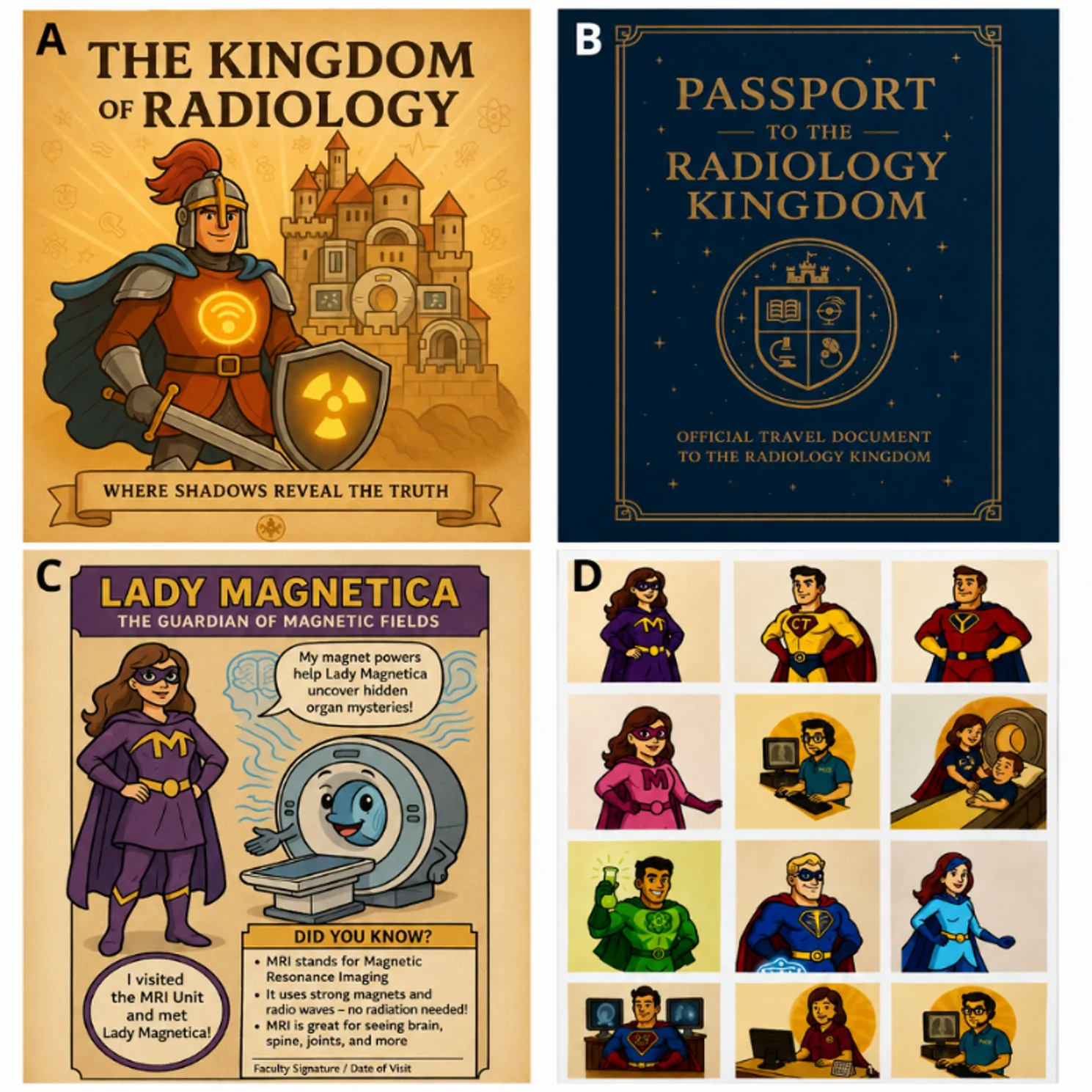
Representative educational materials used in the Radiology Kingdom activity. **A** Cover of the Kingdom of Radiology storybook. **B** Activity passport used to regulate access to the department and document completion of each station. **C** Representative internal page illustrating a themed hero character and corresponding imaging modality. **D** Character-themed sticker set collected during the visit

Each student received three materials (Fig. 1): the Kingdom of Radiology storybook, which introduced six imaging modalities and supporting staff roles through named hero characters in a medieval fantasy setting; the activity passport, used as a structured visit log requiring staff signatures and unit stickers at each station; and a character-themed sticker set. Within the storybook, a clinical case logbook required students to document five real patient examinations during the visit, recording patient ID, accession number, clinical data from the imaging request, modality, and anatomical region.

The clinical case logbook is a component of the educational activity itself, administratively and physically separate from the anonymous research questionnaire analysed in this study. Completed logbooks were returned to the departmental office, stored in a locked cabinet, and reviewed periodically (approximately biweekly to monthly) only by the course organiser to verify completion of the activity; the corresponding visit mark was then recorded in a separate course-assessment record containing the student’s name/ID and mark, without transfer of any patient-level information. Logbooks were subsequently securely destroyed by the course organiser; entries were not accessed by the research team for analysis and were not linked to survey responses, and no patient identifiers, accession numbers, clinical information, or other patient-level information from the logbooks were entered into or analysed as part of the research dataset. Students who wished to retain a memento of the activity were offered a new blank booklet and/or stickers rather than their completed clinical case log. King Saud University Medical City is a teaching hospital in which student clinical observation is a routine and expected part of medical education; as described above, the logbook was used only for internal verification of activity completion, and no patient-identifiable information it contained was extracted, retained, or entered into the research dataset.

Students were briefed before the visit that no tutor would accompany them: navigation would be self-directed using signs, coloured floor trails, and a department map, and the task was to find each unit, complete the logbook case, collect the sticker and signature, and enjoy the process. At each unit, students observed a real patient examination, watched the technologist register patient and examination data, and read the clinical request. At the PACS station, staff demonstrated the archiving system on a large screen. Prior to the academic year, the author conducted briefing sessions with all radiology staff explaining the activity’s objectives and distributing unit-specific character stickers.

### Participants and data quality

Students were eligible for the research questionnaire if they were enrolled in RAD366 during the 2025–2026 academic year and had completed the Radiology Kingdom activity; all third-year students were divided into groups assigned a one-week visit period across the year (November 2025 through April 2026), so eligibility corresponded to the full third-year cohort. The anonymous questionnaire was administered electronically using Google Forms once, at the end of the academic year, after all cohorts had completed their assigned visit and after students had completed their final course examination; each student retrospectively reported the month in which their individual visit had occurred (Table 1). The form did not require sign-in and did not collect names, email addresses, student identification numbers, or other direct participant identifiers; multiple submissions from the same participant were therefore technically possible, and responses were screened for probable duplication as described below. Distribution was through official course channels; participation was voluntary and no incentive was offered. A total of 112 responses were submitted from an eligible population of approximately 270 students, corresponding to an approximate submission rate of 41.5%.

One submission was identified as a probable duplicate, on the basis of an identical Likert response pattern across all items and a submission timestamp approximately ten minutes after another response. Because the questionnaire was anonymous, this identification could not be corroborated against independent identifiers (for example, a unique response token, or matching open-text or demographic responses), and it remains possible that this reflects two students who independently gave identical ratings rather than a true duplicate. This submission was excluded, yielding a final analytic sample of 111 responses (an analytic response rate of approximately 41.1% of eligible students). A post hoc sensitivity analysis was performed by repeating all analyses—item-level, composite, and exploratory subgroup comparisons—with the excluded submission retained (*n* = 112); results are reported in the Results section.

Participant characteristics are summarised in Table 1.

**Table 1:** Participant characteristics (*n* = 111)

Variable	Category	*n*	%
Gender	Male	54	48.6
	Female	57	51.4
Prior radiology exposure	Routine lectures only	89	80.2
	Additional self-directed learning	13	11.7
	Clinical exposure	9	8.1
Month of visit	November	18	16.2
	December	7	6.3
	January	30	27.0
	February	17	15.3
	March	18	16.2
	April	21	18.9

### Questionnaire instrument

The full questionnaire comprised 37 items across seven domains plus open-ended questions. The present study reports on the five-item gamification and instructional-design domain (Items 16–20). Items assessed: the storybook format’s effect on engagement; the sticker system’s effect on active participation; the ID/passport’s effect on overall experience; the Kingdom theme’s effect on memorability; and students’ perceived understanding — the extent to which gamified elements contributed to their understanding of radiology concepts, a self-reported perception rather than an objectively measured learning outcome. All items used a five-point Likert scale (1 = Strongly Disagree, 5 = Strongly Agree). This questionnaire (“Radiology Kingdom Visit: Student Experience and Feedback,” 37 items) was developed specifically for this study and has not been previously published or validated elsewhere; the full English-language instrument is provided as Additional File 1.

Before full implementation, the Radiology Kingdom activity was piloted with a small group of students to evaluate feasibility, workflow, navigation, timing, and logistical procedures. Minor refinements to the activity’s workflow and logistics were made before full implementation. The questionnaire itself, however, did not undergo formal psychometric validation, cognitive interviewing, or expert review before use. This is reported transparently as a limitation (see Discussion).

### Item categorization

The five items were grouped, for descriptive purposes, into a proposed functional categorization: four items correspond to specific design features (Kingdom theme and storybook: narrative-immersive; sticker system: reward-participation; ID/passport: logistical-functional), and one item (gamified understanding) is a global perceived-outcome measure rather than a design feature. This categorization was developed after the statistical analysis, to interpret the observed item-level pattern; it is an interpretive grouping rather than a validated psychometric taxonomy, and the present single-sample data cannot establish its validity (see Limitations).

### Statistical analysis

Descriptive statistics are reported as means and standard deviations, medians and interquartile ranges, and frequencies with percentages. Positive agreement was defined as a score of 4 or 5. A composite score was computed as the unweighted mean of the five items for descriptive and exploratory subgroup comparisons; because the items assess different constructs, it is reported as a secondary summary measure only, with item-level results (Table 2) treated as primary. Cronbach’s alpha was calculated descriptively to assess inter-item internal consistency among the five gamification-related items; because these items assess conceptually distinct aspects of the experience, alpha was not interpreted as evidence of unidimensionality or construct validity, and item-level analyses were treated as primary throughout.

The Shapiro-Wilk test confirmed non-normal distribution (W = 0.709, *p* < 0.001); non-parametric tests were used throughout. Differences across the five items were tested using the Friedman test. Where significant, post-hoc pairwise comparisons were performed using the Wilcoxon signed-rank test with Bonferroni correction (adjusted α = 0.05/10 = 0.005 for ten pairwise comparisons). Between-group comparisons by gender used the Mann-Whitney U test, with effect size reported as the rank-biserial correlation (*r* = 1 − 2U/n₁n₂). For the ten pairwise Wilcoxon comparisons following the significant Friedman test, effect size was reported as the Wilcoxon effect size r (r = Z/√n, where n is the number of non-tied pairs for that comparison and Z is the normal-approximation test statistic), with 95% confidence intervals obtained by percentile bootstrap (5,000 resamples of paired responses; Table 3). Prior exposure and month comparisons used the Kruskal-Wallis H test; η² was additionally reported as an effect size measure for the month-of-visit comparison. Statistical analyses were performed using Python (SciPy version 1.11). A two-tailed p-value < 0.05 was considered statistically significant for primary comparisons; exploratory subgroup comparisons (gender, month, prior exposure) are reported with this caveat throughout the Results.

### Ethical approval

The Radiology Kingdom visits were conducted as a curricular educational activity within RAD366 from November 2025 through April 2026, and students subsequently completed their final course examination on 14 June 2026. Institutional ethics approval for the research evaluation was obtained from the Institutional Review Board of King Saud University Medical City, College of Medicine, King Saud University on 29 June 2026 (Approval No. R26-IRB-510), before administration of the research questionnaire; no questionnaire responses used in this study were collected before this approval. The anonymous questionnaire was distributed only after ethics approval and after completion of students’ final examinations, and included an electronic consent statement; participation was voluntary, and students could decline by not submitting the questionnaire. Because questionnaire administration occurred after completion of the course assessment and final examinations, participation or non-participation in the research questionnaire had no effect on students’ course marks.

## Results

### Item-level results

Cronbach’s α across the five items was 0.841, indicating substantial inter-item correlation; given the heterogeneous constructs represented by the items and the ceiling effects described below, this coefficient was not interpreted as evidence of unidimensionality, construct validity, or a highly reliable measurement scale (see Limitations). The composite score was 4.49 (SD 0.78), median 5.00 (IQR 4.20–5.00); 82.9% of students scored 4 or above. Table 2 presents item-level descriptive statistics.

The Kingdom theme received the highest mean rating (4.63, SD 0.85; 92.8% agreement; 77.5% scoring the maximum of 5). Students’ perceived understanding was rated second (4.60, SD 0.86; 91.0% agreement), followed by the storybook format (4.58, SD 0.86; 90.1% agreement) and the sticker system (4.52, SD 0.96; 87.4% agreement). The ID/passport received the lowest rating (4.12, SD 1.34; 73.0% agreement), with a substantially wider score distribution than the other items and the highest proportion of low scores: 9.9% of students scored 1 and a further 3.6% scored 2.

**Table 2:** Item-level descriptive statistics for the five-item gamification and instructional-design domain (*n* = 111)

Item (Likert 1–5)	Mean (SD)	Median	IQR	*n* ≥ 4 (%)	*n* = 5 (%)
Kingdom theme improved memorability	4.63 (0.85)	5.0	5.0–5.0	103 (92.8)	86 (77.5)
Gamified elements improved understanding	4.60 (0.86)	5.0	5.0–5.0	101 (91.0)	84 (75.7)
Storybook format improved engagement	4.58 (0.86)	5.0	4.0–5.0	100 (90.1)	82 (73.9)
Sticker system improved participation	4.52 (0.96)	5.0	4.0–5.0	97 (87.4)	82 (73.9)
ID/passport improved overall experience	4.12 (1.34)	5.0	3.0–5.0	81 (73.0)	69 (62.2)
Five-item composite (secondary, exploratory)	4.49 (0.78)	5.00	4.20-5.00	92 (82.9)	—

*IQR*  interquartile range. Items ordered by mean score; the composite is a secondary, exploratory summary measure (see Statistical Analysis)

### Friedman test and post-hoc pairwise comparisons

A Friedman test identified a statistically significant difference across the five items (χ²(4) = 32.353, *p* < 0.001). Bonferroni-corrected post-hoc comparisons showed that the ID/passport received significantly lower ratings than all four other items, with large effect sizes (all *p* ≤ 0.001, *r* = 0.61–0.70; Table 3); no significant pairwise differences were detected among the Kingdom theme, gamified understanding, storybook, and sticker system ratings (range of means 4.52–4.63); effect sizes for these six comparisons were small (*r* = 0.03–0.25), and all corresponding 95% confidence intervals crossed zero (Table 3). This pattern is best read as a difference in item ratings rather than confirmed evidence about relative effectiveness, given the items’ differing constructs and the ceiling effects evident in Table 2 (see Limitations). A sensitivity analysis retaining the excluded submission (*n* = 112) produced materially unchanged results: item means shifted by no more than 0.03 points, the Friedman test remained significant (χ²(4) = 32.353, *p* < 0.001), and all ten pairwise comparisons retained the same pattern of statistical significance. Exploratory subgroup comparisons by gender, month, and prior exposure likewise showed no changes in statistical significance, indicating that inclusion of the submission would not have materially altered the study’s principal findings.

**Table 3:** Post-hoc pairwise comparisons following the significant Friedman test (*n* = 111)

Item A	Item B	W statistic	*p*-value	Effect size *r* [95% CI]	Significant (α = 0.005)*
Kingdom theme	ID/passport	87	< 0.001	0.67 [0.44, 0.83]	Yes
Gamified understanding	ID/passport	58	< 0.001	0.70 [0.49, 0.86]	Yes
Storybook	ID/passport	130	< 0.001	0.61 [0.36, 0.80]	Yes
Sticker system	ID/passport	78	< 0.001	0.63 [0.37, 0.83]	Yes
Kingdom theme	Sticker system	127	0.208	0.25 [− 0.17, 0.59]	No
Gamified understanding	Sticker system	190	0.367	0.17 [− 0.21, 0.49]	No
Kingdom theme	Gamified understanding	54	0.435	0.20 [− 0.29, 0.69]	No
Kingdom theme	Storybook	117	0.498	0.14 [− 0.27, 0.53]	No
Storybook	Sticker system	169	0.618	0.10 [− 0.31, 0.45]	No
Gamified understanding	Storybook	240	0.861	0.03 [− 0.32, 0.38]	No

*Bonferroni-corrected significance threshold α = 0.005 (ten pairwise comparisons). Wilcoxon signed-rank test used for all pairs. Exact p-values below 0.001 are reported as returned by the statistical software. Effect size was calculated as the Wilcoxon effect size r (r = Z/√n); 95% confidence intervals were obtained by percentile bootstrap (5,000 resamples)

### Gender differences

An exploratory, non-pre-specified comparison by gender identified a significant difference in the composite score (Female 4.64 vs. Male 4.33; U = 1180, *p* = 0.023, rank-biserial *r* = 0.22) and in three of five items (Kingdom theme *p* = 0.021, storybook *p* = 0.003, sticker system *p* = 0.004; no significant difference for gamified understanding, *p* = 0.237, or the ID/passport, *p* = 0.274; Table 4). Under Bonferroni correction for the five item-level tests (adjusted α = 0.01), the storybook and sticker system comparisons remain significant, while the Kingdom theme comparison does not; these findings are hypothesis-generating (see Limitations).

**Table 4:** Item-level gender comparisons: Mann-Whitney U test (*n* = 111)

Item	*n*	Male Mean (SD)	Female Mean (SD)	U	*p*-value
Kingdom theme	111	4.44 (0.93)	4.81 (0.72)	1139	0.021*
Gamified understanding	111	4.52 (0.88)	4.68 (0.84)	1375	0.237
Storybook engagement	111	4.35 (0.95)	4.79 (0.70)	1104	0.003*
Sticker participation	111	4.31 (1.07)	4.72 (0.80)	1147	0.004*
ID/passport experience	111	4.02 (1.42)	4.21 (1.27)	1393	0.274
Five-item composite (secondary)	111	4.33 (0.84)	4.64 (0.68)	1180	0.023*

*p < 0.05 (uncorrected; see text for Bonferroni-adjusted threshold). Male *n* = 54, Female *n* = 57. Gender comparison was not pre-specified; findings are exploratory. Effect size reported as rank-biserial correlation, *r* = 1 − 2U/n₁n₂

### Month of visit and prior exposure

No significant effect of month of visit (Kruskal-Wallis H = 6.976, *p* = 0.222, η² = 0.019) or prior radiology exposure (H = 0.071, *p* = 0.965) on the composite score was identified; month subgroups were small and uneven (7–30 students), which limits power to detect a true difference (see Limitations).

## Discussion

### Principal findings

This study examined how medical students rated five items in the gamification and instructional-design domain of a post-visit questionnaire. The ID/passport received significantly lower ratings than the other four items, which did not differ significantly from one another. Female students rated the composite and three of five items higher than male students, an exploratory finding. Ratings did not differ by visit month or prior radiology exposure.

### Interpretation and comparison with prior literature

Rather than indicating simply that students enjoyed the activity, these ratings speak to two different things: engagement with the activity’s design features (storybook, sticker system, Kingdom theme) and a perceived learning outcome (gamified understanding), the latter a global outcome measure distinct from the four design-feature items (see Item Categorization). That the perceived-outcome item was rated similarly highly to the highest-rated design-feature items suggests students perceived the gamified format as contributing to their conceptual understanding of radiology concepts, not merely as an enjoyable addition — though this remains a self-reported perception rather than a demonstrated learning gain. The pattern across all five items is also consistent with self-directed and experiential learning theory, in which active, independently-navigated engagement with an authentic environment, rather than passive instruction, is proposed as a precondition for learning [8, 9], and with prior reports that game-design elements aligned with learning objectives are positively received by learners [2, 3]. Most published gamification evaluations report a single overall score; this study’s item-level comparison shows that even one gamified activity can elicit systematically different ratings across its components [4], though it does not establish which underlying item property causes the differences observed, since the items differ in wording and construct. Recent quasi-experimental studies specifically evaluating gamified radiology instruction have reported favourable findings in knowledge or performance outcomes and student perceptions following gamified interventions [10, 11], and a Delphi study among medical educators has begun to identify and prioritise which specific gamification elements are considered most valuable [12], a question this item-level analysis speaks to directly by showing that ratings can differ significantly even within a single gamified activity. Female students reported higher ratings on the composite score and on three individual items; however, this subgroup comparison was exploratory and not based on a pre-specified hypothesis, and the present study did not measure the mechanisms (for example, narrative or social-reward engagement) that might explain such a difference. Although correction for multiple item-level comparisons was applied, the modest effect size of the composite-score difference (*r* = 0.22) suggests limited practical separation between groups, and no conclusions can be drawn regarding gender-specific responses to particular gamification features; this finding should be considered hypothesis-generating and requires confirmation in appropriately designed studies.

### Interpretation of the passport findings

Several non-exclusive explanations may account for the ID/passport’s lower and more variable ratings: the item asked about “overall experience,” a broader outcome than the other items’ specific engagement, participation, or memorability outcomes; students may have perceived the passport primarily as a logistical or access-control mechanism rather than as part of the narrative; its capped availability may have affected some students’ experience; or other aspects of the mechanic may have been less intuitive or engaging than the narrative and reward elements. These explanations are hypothesis-generating only, were not directly assessed by the questionnaire, and cannot be distinguished using the present data; the lower item-level rating should not be interpreted as evidence that the passport was educationally less effective than the storybook, sticker system, or Kingdom theme (see Limitations).

### Practical implications

Several operational choices may have contributed to the intervention’s consistently high ratings and its successful integration into a busy clinical service across a full academic year. The passport system was designed to address the crowd-management problem of placing an entire year-group inside a working department: capping simultaneous presence at 15, without a fixed schedule, protected clinical workflow while giving every student flexible, self-paced access across the year. Administering the questionnaire once, after all cohorts finished, rather than assessing each visit individually, reduced ongoing administrative burden.

Running the activity required modest resources: one-time authoring of a themed storybook and character set; a small recurring stock of passports and stickers, issued and collected by existing administrative staff rather than dedicated study personnel; brief annual staff briefings at each station; and signage enabling self-directed navigation, removing the need for a tutor to accompany each group. No specialised software, extra clinical time, or dedicated teaching staff were required beyond the author’s initial design effort.

This structure could be adapted by other departments or medical schools seeking to introduce low-cost, self-directed clinical immersion without major curricular change: a narrative frame suited to the local context, an access mechanic sized to real capacity constraints, a structured logbook requiring documented completion at each station, and signage-supported self-directed navigation. The specific theme may be less important for replication than these structural features—capped access, structured task completion, and staff awareness—which represent practical components that could be adapted to local settings.

Two lessons are worth recording for institutions replicating this design. First, the passport’s lower rating suggests a logistical component should be evaluated, and if necessary redesigned, separately from narrative and reward components rather than assumed to succeed on the same visual theme. Second, a single year-end questionnaire is administratively convenient but means visit experiences are recalled retrospectively, sometimes months later; institutions prioritising item-level detail may prefer more frequent, visit-linked data collection.

### Limitations

Several limitations affect interpretation. This was a single-centre, uncontrolled survey with no comparison group or baseline, so no causal claims can be made. The analytic response rate of approximately 41.1% introduces an important possibility of non-response bias: students with particularly positive or memorable experiences may have been more likely to participate, potentially contributing to the high observed ratings. Because the questionnaire was anonymous, respondent status could not be linked to identifiable academic or demographic records, and a direct comparison between respondents and non-respondents was therefore not possible; the findings represent the perceptions of respondents and should not be assumed to represent those of the entire eligible cohort. The questionnaire was administered once, after completion of the course assessment period, rather than immediately after each visit; because visits occurred from November 2025 through April 2026, the recall interval varied substantially across participants, and students who visited earlier may have recalled aspects of the experience less accurately than those who visited later, particularly for items concerning memorability and overall experience. Although no statistically significant differences were detected across visit months, the month-specific groups were small and uneven (7–30 students); this exploratory analysis therefore cannot exclude an effect of visit timing, and the absence of significance should not be interpreted as evidence that timing had no effect. Future evaluations should consider administering questionnaires prospectively at a standardised interval after each visit. The author designed the activity, serves as course organiser for RAD366, and evaluated the resulting data alone; although the visit itself was self-directed and unsupervised, with no tutor present, students’ awareness that the activity’s designer would also see the evaluation results creates a risk of allegiance and social-desirability bias. The questionnaire underwent no formal psychometric validation, cognitive interviewing, or pilot testing, despite the educational activity itself being piloted before implementation (see Methods); its interpretive validity therefore remains unconfirmed. The one excluded submission was a probable, not confirmed, duplicate; however, a sensitivity analysis retaining it produced materially unchanged results (see Results).

The five items do not measure a common construct — memorability, understanding, engagement, participation, and overall experience — and only four correspond to a design feature, the fifth (students’ perceived understanding) being a global outcome judgement. This is the central caveat: rating differences may reflect item wording as much as design-feature effectiveness, limiting how strongly the ID/passport finding generalises. Substantial ceiling effects were present, with most items exceeding 85% positive agreement (range 87.4–92.8%) and a median of 5 for all five items; this restricted variability limits the questionnaire’s ability to discriminate among different degrees of positive perception and may reduce sensitivity to differences among the more highly rated items. Consequently, non-significant differences — including the absence of a significant effect of prior radiology exposure, for which the clinically-exposed subgroup was small — should not be interpreted as evidence of equivalence between groups. Cronbach’s α should likewise be interpreted cautiously in the presence of both ceiling effects and heterogeneous item constructs and does not establish unidimensionality, validity, or reliability of the instrument as a measurement scale. All outcomes are self-reported perceptions with no objective knowledge, behavioural, or retention measure — the study supports statements about student-reported perceptions, not objectively demonstrated learning or educational effectiveness; in particular, the item addressing whether gamified elements “improved understanding of radiology concepts” reflects students’ perceived understanding and should not be interpreted as evidence of objectively measured knowledge acquisition.

The proposed item categorization is an interpretive framework, not a validated taxonomy, and cannot be established by this single-sample dataset. The gender, month, and prior-exposure comparisons were exploratory, not pre-specified, and only partially robust to multiplicity correction; subgroups were small and uneven, limiting power. No qualitative data were analysed, limiting the ability to explain why students rated the ID/passport as they did.

### Future research

Future controlled, prospective studies comparing this intervention with conventional radiology department exposure — incorporating pre- and post-intervention knowledge assessment, delayed retention testing, and, where feasible, objective structured assessment — are needed before conclusions regarding educational effectiveness can be drawn. A delayed knowledge-retention study, ideally with a non-gamified comparison arm, would test whether the character-naming scheme functions as an effective mnemonic device, as elaborative encoding theory predicts [6, 13]. Qualitative work on the ID/passport rating, and a study pre-registering gender and visit timing with adequate power, would clarify whether these patterns generalise. Future work should also include formal development and validation of an instrument specifically designed to evaluate gamified clinical and radiology educational experiences.

## Conclusion

Students reported highly positive perceptions of the storybook, Kingdom theme, sticker system, and gamified elements’ perceived contribution to their understanding (all item means ≥ 4.52/5), whereas the ID/passport—the activity’s organisational and access-control component—received significantly lower ratings than the other four items. Because these items assessed different constructs, this finding should not be interpreted as evidence that the passport was educationally less effective than the other intervention elements; possible explanations for the lower rating are discussed above as hypotheses that the present data cannot distinguish.

This study demonstrates the feasibility of implementing a resource-modest, narrative-based, self-directed clinical immersion activity — incorporating a storybook, a capped-access passport, and self-directed navigation — within a busy radiology department. Students reported highly positive perceptions of its narrative and engagement-oriented elements; however, the uncontrolled observational design does not establish that the intervention itself caused improvements in engagement, understanding, memorability, or learning. The passport mechanic was designed to address crowd management in a working department, while the narrative and reward elements received consistently high student ratings throughout the academic year. For institutions seeking to provide structured, low-supervision exposure to a clinical department’s multidisciplinary team, the Radiology Kingdom offers a practical template that may be adapted to similar educational settings. The Radiology Kingdom therefore represents a practical educational model that warrants evaluation in controlled, multi-institutional studies incorporating objective measures of learning and retention before conclusions regarding educational effectiveness or scalability can be drawn.

## Supplementary Information

Supplementary Material 1.

## Data Availability

Anonymised aggregate data are available from the corresponding author upon reasonable request.
