# Supplementary material for: Medical students’ perceptions of gamified design elements in a self-directed radiology department visit
Source: BMC Med Educ. 2026 Sep 24;26:1654. doi: 10.1186/s12909-026-10460-y (PMC13670702; doi:10.1186/s12909-026-10460-y)
Supplement: Supplementary file 1 [file 12909_2026_10460_MOESM1_ESM.docx]

**Additional File 1**

**Radiology Kingdom Visit: Student Experience and Feedback Questionnaire (English version)**

Companion manuscript: “Medical Students’ Perceptions of Gamified Design Elements in a Self-Directed Radiology Department Visit.”

Instrument development: This questionnaire was developed specifically for this study by the study team; it has not been previously published or validated elsewhere. It was administered electronically via Google Forms to medical students following completion of the self-directed Radiology Kingdom department visit.

Format: The instrument comprises 37 items across three demographic items (multiple choice), 31 attitudinal items rated on a 5-point Likert scale (1 = Strongly Disagree to 5 = Strongly Agree), and 3 open-text items. Items are grouped below under the section headers used in the original instrument.

*Note: Response options for the demographic items (Items 1–3) are reproduced here as free-text/category questions.*

**Section: Participation (demographics)**

| **#** | **Item** |
| --- | --- |
| 1 | What is your gender? |
| 2 | Before this visit, how would you describe your radiology exposure? |
| 3 | In which month did you complete your visit? |

**Section: Overall Experience (5-point Likert: Strongly Disagree – Strongly Agree)**

| **#** | **Item** |
| --- | --- |
| 4 | The visit was enjoyable |
| 5 | The visit was engaging and interactive |
| 6 | Overall, I am satisfied with the visit |

**Section: Educational Value (5-point Likert)**

| **#** | **Item** |
| --- | --- |
| 7 | I gained a better understanding of how a radiology department operates |
| 8 | I gained a better understanding of patient flow within the radiology department |
| 9 | The visit helped me connect theoretical knowledge with real clinical practice |

**Section: IPE and Teamwork Perception (5-point Likert)**

| **#** | **Item** |
| --- | --- |
| 10 | I gained a better understanding of the role of radiology technologists |
| 11 | I gained a better understanding of the role of radiology nurses |
| 12 | I gained a better understanding of the role of PACS/technical staff |
| 13 | I gained a better understanding of how healthcare professionals collaborate within the radiology department |
| 14 | The visit increased my appreciation of teamwork in healthcare |
| 15 | The visit improved my understanding of the importance of communication between healthcare professionals |

**Section: Instructional Design / Gamification (5-point Likert)**

| **#** | **Item** |
| --- | --- |
| 16 | The storybook format improved my engagement during the visit |
| 17 | The sticker system improved my active participation |
| 18 | The ID/passport improved my overall experience |
| 19 | The “Radiology Kingdom” theme improved the memorability of the visit |
| 20 | The gamified elements improved my understanding of radiology concepts |

**Section: Organization, Logistics and Learning Structure (5-point Likert)**

| **#** | **Item** |
| --- | --- |
| 21 | The instructions and overall process of the visit were clear |
| 22 | The ID/passport system facilitated the organization of the visit |
| 23 | The number of students was appropriate for a comfortable learning environment |
| 24 | The visit design allowed easy navigation across different radiology units |
| 25 | The visit structure allowed students to complete all required activities |
| 26 | The self-directed (flexible) structure of the visit was suitable for my learning |
| 27 | The one-week period provided to complete the visit was sufficient |

**Section: Interaction with Department Staff and Learning Environment (5-point Likert)**

| **#** | **Item** |
| --- | --- |
| 28 | I felt comfortable interacting with staff during the visit |
| 29 | Department staff were welcoming and supportive during the visit |
| 30 | The explanations provided by staff supported my learning |

**Section: Overall Impact on Perception, Interest, and Confidence (5-point Likert)**

| **#** | **Item** |
| --- | --- |
| 31 | This visit improved my overall perception of the field of radiology |
| 32 | This visit increased my interest in radiology |
| 33 | The visit improved my confidence in navigating a radiology department environment |
| 34 | I would recommend continuing this activity for future students |

**Section: Open Feedback (free text)**

| **#** | **Item** |
| --- | --- |
| 35 | What was the most valuable part of the visit, and why? (open text) |
| 36 | What improvements would you suggest for this activity in the future? (open text) |
| 37 | Any additional comments? (open text) |

*End of instrument.*
